# The bacterial species profiles of the lingual and salivary microbiota differ with basic tastes sensitivity in human

**DOI:** 10.1038/s41598-023-47636-1

**Published:** 2023-11-20

**Authors:** Hélène Licandro, Caroline Truntzer, Sébastien Fromentin, Christian Morabito, Benoit Quinquis, Nicolas Pons, Christophe Martin, Hervé M. Blottière, Eric Neyraud

**Affiliations:** 1 UMR A 02.102 Procédés Alimentaires et Microbiologiques (PAM), Institut Agro Dijon, Université de Bourgogne Franche-Comté, 21000 Dijon, France; 2 Plateforme de Transfert en Biologie Cancérologique, Georges François Leclerc Cancer Center – UNICANCER, 1 rue du Professeur Marion, 21000 Dijon, France; 3 UMR INSERM 1231, 7 Boulevard Jeanne d’Arc, 21000 Dijon, France; 4 MetaGenoPolis, INRAE, AgroParisTech, Université Paris-Saclay, Paris, France; 5 Centre des Sciences du Goût et de l’Alimentation, Institut Agro Dijon, CNRS, INRAE, Université de Bourgogne, Université de Bourgogne Franche-Comté, 21000 Dijon, France; 6 PROBE Research Infrastructure, Chemosens Facility, 21000 Dijon, France; 7 INRAE, UMR 1280, PhAN, Nantes Université, 44000 Nantes, France

**Keywords:** Metagenomics, Microbiome

## Abstract

Taste perception is crucial and impairments, which can be linked to pathologies, can lead to eating disorders. It is triggered by taste compounds stimulating receptors located on the tongue. However, the tongue is covered by a film containing saliva and microorganisms suspected to modulate the taste receptor environment. The present study aimed to elucidate the links between taste sensitivity (sweetness, sourness, bitterness, saltiness, umami) and the salivary as well as the tongue microbiota using shotgun metagenomics. 109 bacterial species were correlated with at least one taste. Interestingly, when a species was correlated with at least two tastes, the correlations were unidirectional, indicating a putative global implication. Some *Streptococcus*, *SR1* and *Rickenellaceae* species correlated with five tastes. When comparing both ecosystems, saliva appears to be a better taste predictor than tongue. This work shows the implication of the oral microbiota in taste and exhibits specificities depending on the ecosystem considered.

## Introduction

As taste plays a key role in the perception of food and consequently influences food choice, it is associated with the avoidance of potentially poisonous molecules, which usually taste bitter, or the attraction towards energy-rich foods, for example, those tasting sweet. Therefore, impairments in this sensation, which can be linked to pathologies, can lead to eating disorders and malnutrition^1^. Taste perception is the sensation elicited by taste molecules when they stimulate the tongue's taste receptors, and it is admitted that five basic tastes exist, sweet, salty, sour, bitter and umami. Taste perception varies strongly between individuals^2, 3^, and the originating factors of this variability are incompletely understood. If polymorphisms in genes involved in taste perception contribute to this variability^4^, other factors linked to the oral environment, such as saliva, could also contribute to the modulation of this perception^5, 6^.

Anatomically, the tongue is a muscle covered by a specialized mucosa with irregular topography. This structure contains numerous depressions and constitutes an ecological site favourable to the accumulation of saliva, food and cellular debris and microorganisms^7^. Therefore, the lingual mucosal surface is covered by a biological film comprising desquamating cells, resting saliva and microorganisms; some studies have established relationships between the composition of this film and taste perception^8, 9^. The main hypotheses explaining these relationships are that this film could act as a physical barrier that limits the access of high molecular weight taste molecules, such as bitter compounds, to taste receptors^10^ or that bacterial metabolism could modulate taste perception. This last phenomenon includes the use of sugars and amino acids as substrates and the production of organic acids affecting sweet, umami and sour sensations^7, 11^.

Recently, the implication of the tongue microbiota has gained interest, and several groups have tried to establish links between it and taste perception, aided by the increasing accessibility of sequencing techniques, such as 16S rRNA gene metabarcoding. It is difficult to determine the specific implication of the tongue microbiota in basic taste perception; some authors focused on the specific fat taste in obese subjects^12, 13^, and others on the global taste sensitivity elicited by the bitter PROP (6-*n*-propylthiouracil)^14^. Recently, two studies revealed contradictory findings regarding the relationships between the basic tastes and the oral microbiota^15, 16^; Cattaneo et al.^15^ found association between tongue dorsum microbiota and gustatory functions whereas Fluitman et al.^16^ did not. It can be concluded from these previous works that the relationships between the tongue microbiota and the different basic tastes are complicated to resolve. One possible explanation is the lack of precision of the 16S rRNA gene sequencing that provides information at the genus level when specific species or subspecies are involved. Another hypothesis is that the tongue dorsum is not the best site to assess. Indeed, whole saliva appears a more interesting fluid for analysis than particular oral sites, especially as it is easy to sample and has potential as a biomarker source^17^. In addition, the bacterial composition of saliva is well-characterized in comparison with other oral niches^18^. Some researchers have focused on salivary microbiota and taste perception^19, 20^ and observed differences in the ability to correctly identify different taste qualities depending on the abundance of Bacteroidota^19^ or with taste-preference gene polymorphisms^20^. Others observed association between taste compound intake^21^ or food allergy^22^ and salivary microbiota composition. However, only one study^23^ investigated both lingual and salivary microbiota and highlighted differences in the associations between taste and lingual and salivary microbiota according to the site. A specific positive association between bitterness and Bacteroidota was observed in the lingual film, whereas a negative association between Actinobacteriota and saltiness was observed when focusing on saliva. However, the targeted method used, qPCR of the phyla, did not provide information on the specific genera or species.

Thus, the objective of the present work was to elucidate whether sensitivity to the five primary tastes is linked to oral microbiota in human with a special focus on the microbiota of the tongue dorsum and whole resting saliva microbiota. Shotgun metagenomics, which allows identification of species was used to investigate these relationships in greater depth than previous studies performed using 16S rRNA gene sequencing.

## Methods

### Subject selection

One hundred subjects, 50 females and 50 males aged 20 to 64 (mean: 41 ± 14) years were recruited. The exclusion criteria were as follows: pregnant women; smokers, those with food allergies; those who had been taking medication longer than one month; those who underwent previous radiotherapy of the head or neck; those using antibiotics, undergoing dental treatment or using antiseptic mouthwash in the preceding month; and individuals with Sjögren syndrome, lupus, dermatomyositis, lichen planus, Behçet, oral mycosis, gingivitis, gingivostomatitis, recurrent aphthae, and inflammatory bowel diseases.

### Taste sensitivity determination and biological sampling

Participants were required to visit the laboratory twice. They were instructed not to brush their teeth, eat or drink anything other than water at least 2 h before sample collection, which occurred between 9 and 11 a.m. During the first visit, a sample of unstimulated saliva was collected in a container on ice by passive drooling for 5 min. For participants who did not secrete a minimum of 2 ml, extra time was allowed until they reached this volume. After a short rest, tongue film was collected as follows: participants swallowed saliva, immediately stuck their tongue out as far as possible, and maintained this pose while the film was collected by scraping the tongue with a plastic stick from the root to the apex in a single scraping motion. The same experimenter performed all the sampling to ensure repeatability. The saliva and tongue film samples were frozen at 80 °C immediately and stored until the analyses were conducted. After a short rest, taste sensitivity for the five basic tastes, sweet (fructose), salty (sodium chloride), sour (citric acid), bitter (quinine hydrochloride), and umami (monosodium glutamate), was determined using the T@sty test™ (Patent WO2015/165880) following the procedure described in Martin and Neyraud^24^. Briefly, the T@sty test is presented in the form of several edible test sheets, each allowing for one given taste sensitivity measurement. Each test sheet consists of six triplets of detachable precut discs (wafer paper) that need to be placed on the first third of the tongue. The triplets all consist of one disc containing the taste substance and two neutral discs. The taste compounds, dissolved in deionized water, were printed on the surface of the discs using a food-grade inkjet printer. The position of the tasty disc varies randomly depending on the triplets and the test sheets. The surface concentration of the taste compound and the intensity of taste gradually increases from the first to the sixth triplets. Distilled water is printed in the same manner on the neutral discs to make them look the same as the tasty discs. For each test sheet, and thus for each taste, the subjects had to successively evaluate the six triplets, starting with the triplet containing the lowest concentration located at the top of the sheet and then moving downwards.

The measured scores vary from 1 to 6 depending on the last triplet where the subject detect the taste concentration. A high score signifies a high taste sensitivity. At the end of the visit, a T@sty test™ kit was given to the participants, who conducted the test at home before the second visit. The second visit occurred approximately 2 weeks after the first and the same procedures were conducted. Overall, 2 unstimulated saliva and tongue film samples and 3 repeat T@sty test™ data were obtained for all participants.

### DNA extraction and shotgun sequencing

Saliva samples were thawed and centrifuged at 17,000 ×*g* for 10 min. DNA from both saliva pellets and lingual film samples was extracted following the publicly available MGP SOP 002 V1 protocol (https://mgps.eu/standard-operating-procedure/) inspired by the International Human Microbiome Standards (IHMS) guidelines and adapted for low-biomass samples. DNA was quantitated using Qubit Fluorometric Quantitation (ThermoFisher Scientific, Waltham, US) and qualified using DNA size profiling on a Fragment Analyser (Agilent Technologies, Santa Clara, US). A total of 422 ± 300 ng of high molecular weight DNA (> 10 kbp) was used to build the library. Shearing of DNA into fragments of approximately 150 bp was performed using an ultrasonicator (Covaris, Woburn, US), and DNA fragment library construction was performed using the Ion Plus Fragment Library and Ion Xpress Barcode Adapters Kits (ThermoFisher Scientific, Waltham, US). Purified and amplified DNA fragment libraries were sequenced using the Ion Proton Sequencer (ThermoFisher Scientific, Waltham, US), generating 22.1 million reads ± 4.3 of 150 bp (on average) per sample.

### Microbial gene count table

Reads were cleaned using ALIENTRIMMER v0.4 to (i) remove resilient sequencing adapters and (ii) trim low-quality nucleotides at the 3’ side using a quality and length cutoff of 20 and 45 bp, respectively. Cleaned reads were subsequently filtered using a human reference sequence (human genome version: Homo_sapiens_chm13_t2t_v1_1) with an identity score threshold of 90%. Gene abundance profiling was performed using the 8.4 million gene catalogue of the human oral microbiome [https://doi.org/10.15454/WQ4UTV]. Filtered high-quality reads were mapped with an identity threshold of 95% to the 8.4 million-gene oral catalogue using Bowtie2 (v2.3.5) included in METEOR v3.2 software (https://forgemia.inra.fr/metagenopolis/meteor). A gene abundance profiling table was generated through a two-step procedure using METEOR v3.2. First, reads mapped to a unique gene in the catalogue were attributed to their corresponding genes. Second, reads that mapped with the same alignment score to multiple genes in the catalogue were attributed according to the ratio of their unique mapping counts. The gene abundance table was processed for rarefaction, normalization, and further analysis using the MetaOMineR (momr, v1.31) R package. Read counts were rarefied to decrease technical bias due to different sequencing depths and avoid any artefacts of sample size on low abundance genes. The gene abundance table was rarefied to 2 million reads per sample by random sampling of 2 million mapped reads without replacement. The resulting rarefied gene abundance table was normalized according to the fragments per kilobase per million mapped reads (FPKM) approach to give the gene abundance profile table.

### Metagenomic species pangenome (MSP) profiles

Metagenomic species pangenomes (MSPs) are coabundant gene groups corresponding to microbial species. A total of 853 MSPs were clustered using MSPminer^25^. MSP abundances were estimated as the mean abundance of the 100 genes defining a robust centroid of the cluster (if more than 10% of these genes demonstrated positive signals). MSP richness (MSP count) was calculated directly from the rarefied MSP abundance matrix. Bacterial gene richness (gene count) was calculated by counting the number of genes detected at least once in a given sample, using the average number of genes counted in 10 independent rarefaction experiments. Finally, an occurrence filter was used, leaving 666 species present in at least one sample.

### Data analysis

A principal component analysis (PCA; biplot representation) of the covariance matrix provided a multidimensional representation of the links between the scores obtained for the different tastes. The participants were represented using different symbols depending on their age and sex. A second PCA was performed on saliva and lingual film species to depict the dataset variability. By correlating principal components to descriptive variables, such as age, sex and salivary flow, we aimed to evaluate whether species-major information is linked to these participants characteristics. Spearman correlation tests were used to evaluate the significance of correlations.

Correlations between phylum, genus and species expression quantified in saliva and lingual film were tested for each of the 5 tastes using the Spearman correlation test. *p* values < 0.05 were considered statistically significant. Features significantly correlated to at least one taste were selected and depicted through heatmaps showing significant Spearman correlation values. A positive correlation means that a higher abundance of the microbial taxa corresponds to a higher sensitivity to the taste. We chose to perform selection based on raw p values rather than FDR-adjusted *p* values to allow a wider selection of entities of interest.

Classifier models were built with each taste score using saliva species abundancies or lingual species abundancies as the input to predict whether each subject was “high” or “low” in this specific taste. “High” and “low” statuses were defined using the median value of the taste score. Participants with taste scores higher than the median were defined as “high” for this specific taste, and those with lower scores were defined as “low”. The classifiers were PLS DA (discriminant analysis) models built on a training set comprising 80% of the participants and tested on a test set of participants (the remaining 20%). This data split (training and test sets) was reproduced a hundred times to remove any potential bias due to the randomized splitting between two sets. With each data splitting, the AUC (Area Under the Receiver Operating Characteristics) on the test set was used as predictive score to determine classifier performances. An excellent model has AUC near to 1 whereas a poor model has an AUC near 0. When AUC is 0.5, the model has no class separation capacity. The CARET R package was used to compute these classifier models (https://CRAN.R-project.org/package=caret).

**Figure 1:**
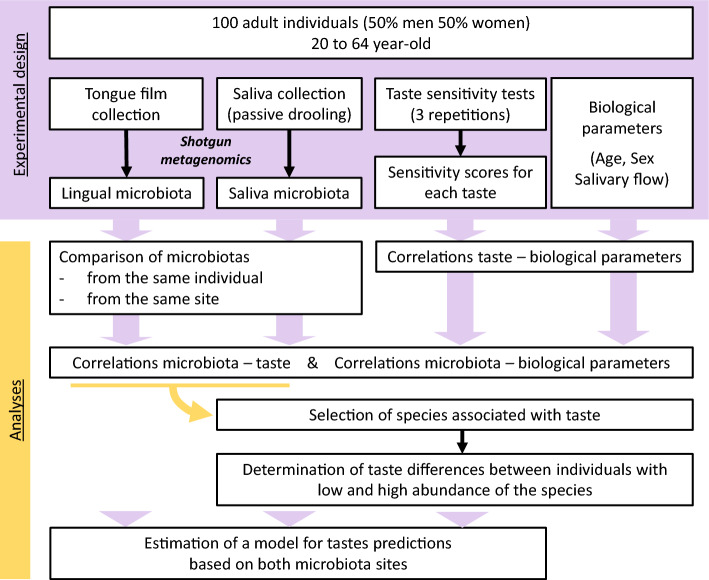
Flow chart of the different steps of the study.

A flow chart of the different steps of the study is presented Fig. 1.

### Ethics approval and consent to participate

Written informed consent was obtained from each participant in the study. The protocol was approved by the Ethics Committee: Comité de Protection des Personnes Ouest 5 (CPP no. 2016-A01954-47).

## Results

### Taste sensitivity and subject characteristics

**Figure 2:**
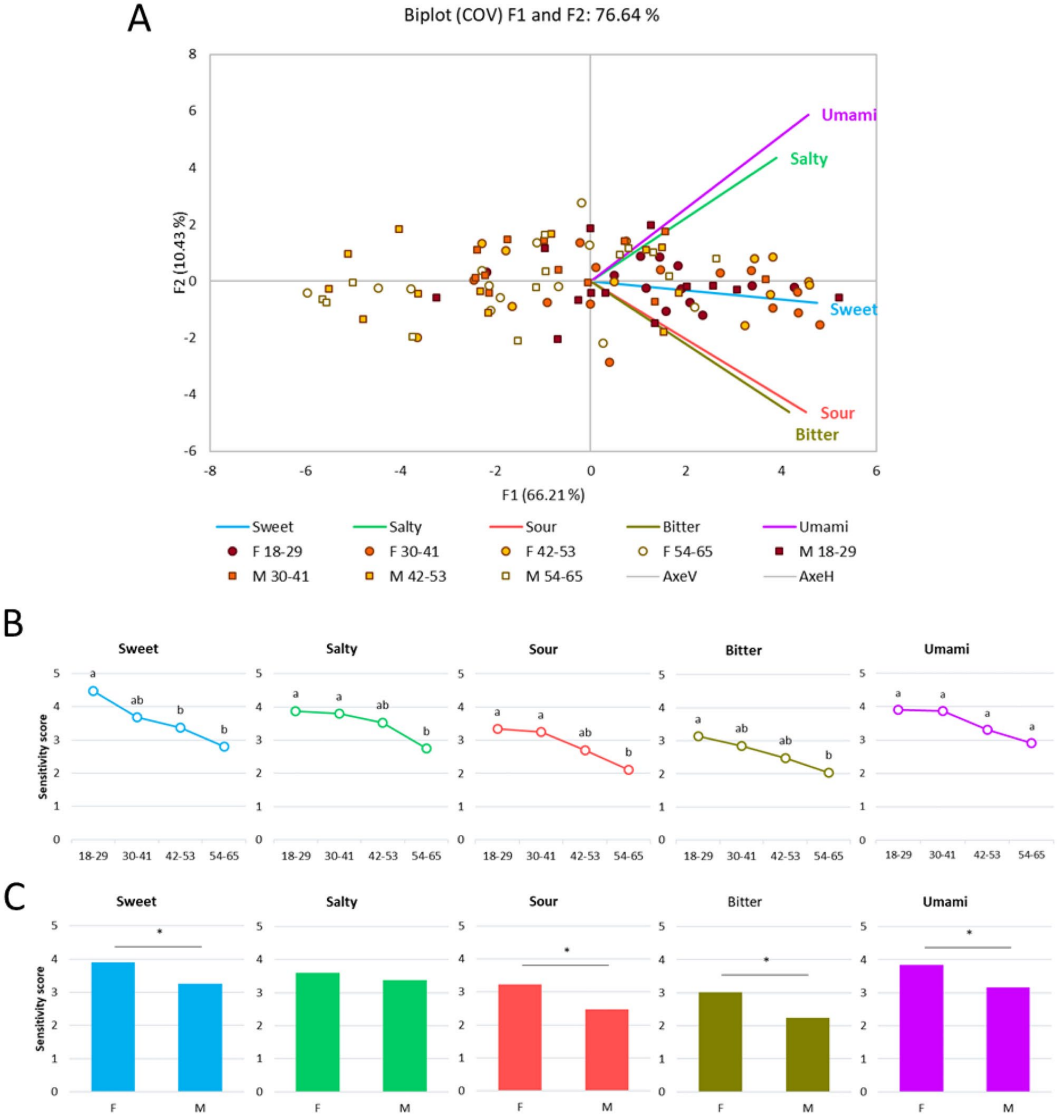
Taste sensitivity of the panel (**A**) Biplot representation of the distribution of the participants (N = 100). Each dot represents the average of 3 tests. Females are represented by circles, and males by squares. The intensity of colour decreases with age. (**B**) Average taste sensitivity scores for 4 age classes (N = 25). Different letters indicate significant differences (*p* ≤ 0.05) between the age groups for a given taste. (**C**) Average sensitivity scores for males (N = 50) and females (N = 50). A star indicates a significant difference (*p* ≤ 0.05) between groups. A high score signifies a high taste sensitivity.

Subjects displayed heterogeneous sensitivity to the different tastes, as shown in Fig. 2A. Taste sensitivity for sourness appears linked with bitterness and umami with saltiness. The sensitivity scores associated with the different taste modalities decrease significantly with age (Fig. 2B), and a significant sex effect was observed for all taste modalities except saltiness, with females being more sensitive than males (Fig. 2C).

### Saliva and lingual film microbiota

**Figure 3:**
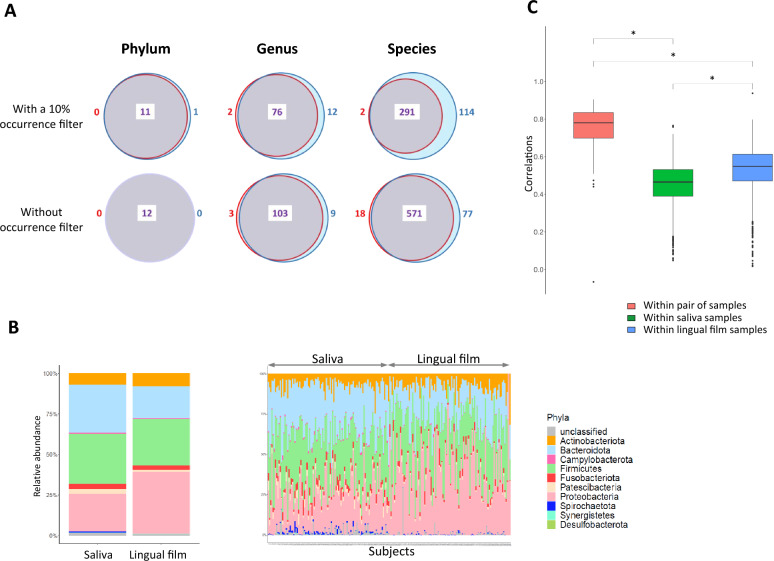
Bacterial composition of saliva and lingual film. (**A**) Venn diagrams showing the number of features (resp. phylum, genus and species) unique to the saliva (in blue) or lingual film (in red) and common to both (in purple), with (above) or without (below) occurrence filtering. (**B**) Relative abundance of the different phyla in the saliva and lingual film at the group level on the left (relative abundance average) and at the individual level on the right. (**C**) Box plot representation of the Spearman correlation coefficients. The green boxplot displays correlations between saliva samples; the blue box displays correlations between lingual samples, and the red box displays correlations between saliva and lingual samples from the same subject. A star indicates a significant difference (*p* ≤ 2.22–16) between samples.

A total of 666 microbial species were identified in the saliva and lingual film samples (Fig. 3A). Most of these species (86%) were common to both oral habitats. The number of nonshared species was slightly higher in saliva than in lingual film (12% and 3%, respectively), and the same observations were obtained at the genus level (8% and 3%, respectively), for a total of 115 genera. The 12 phyla identified are common to saliva and lingual film. Interestingly, when restricting the analysis to the species present in at least 10% of individuals (application of a 10% occurrence filter on each body site, giving 405 species in the saliva and 293 species in the lingual film), the proportion of shared species between the two ecosystems is lower, with almost all the nonshared species identified in the saliva (114 species specific to saliva and 2 specific to lingual film). The same observations were observed to a lesser extent for genera, but only one phylum was specific to saliva. This finding shows that the most prevalent species are not always found in the lingual film, while the less prevalent species are most often found in these samples. In addition to species richness, the relative abundance of some phyla varied between the two different ecosystems. This is illustrated at the individual and group levels, where a higher abundance of Bacteroidota and a lower abundance of Proteobacteria can be observed in saliva compared to that in lingual film (Fig. 3B). Moreover, approximately 90% of the species whose relative abundance significantly differed between the saliva and tongue dorsum were more abundant in the saliva (270 species vs. 29 species, Supplementary Table S1). Notably, there was a significantly higher correlation between lingual and saliva samples from the same participants than among samples from different participants within the same ecosystem (R = 0.75 between 2 samples of the same subjects; R = 0.46 between samples from the salivary ecosystem and R = 0.53 between samples from the lingual ecosystem). This finding points towards a stronger heterogeneity of the microbiota between the same ecosystems in different participants than for both ecosystems within the same subject (Fig. 3C). Regarding the characteristics of the subjects, neither salivary nor lingual microbiota was affected by sex; however, the lingual microbiota was significantly affected by age, and the salivary microbiota was affected by the resting salivary flow rate (Table 1).

**Table 1:** Estimation of Spearman correlation coefficients between coordinates of samples on first and second components of Principal Component Analysis based respectively on saliva and lingual film species, and age and salivary flow.

	Saliva		Lingual film	
	Axis 1 (9%)	Axis 2 (6%)	Axis 1 (15%)	Axis 2 (9%)
Age	− 0.07	− 0.12	0.31**	− 0.09
Salivary flow	0.24*	0.35***	− 0.04	0.04

Between brackets: percent of variance explained by correspondent principal axis.

*p < 0.05; **p < 0.01; ***p < 0.001.

Significant values are in [bolditalics].

### Relationships between taste sensitivity and oral microbiota

**Figure 4:**
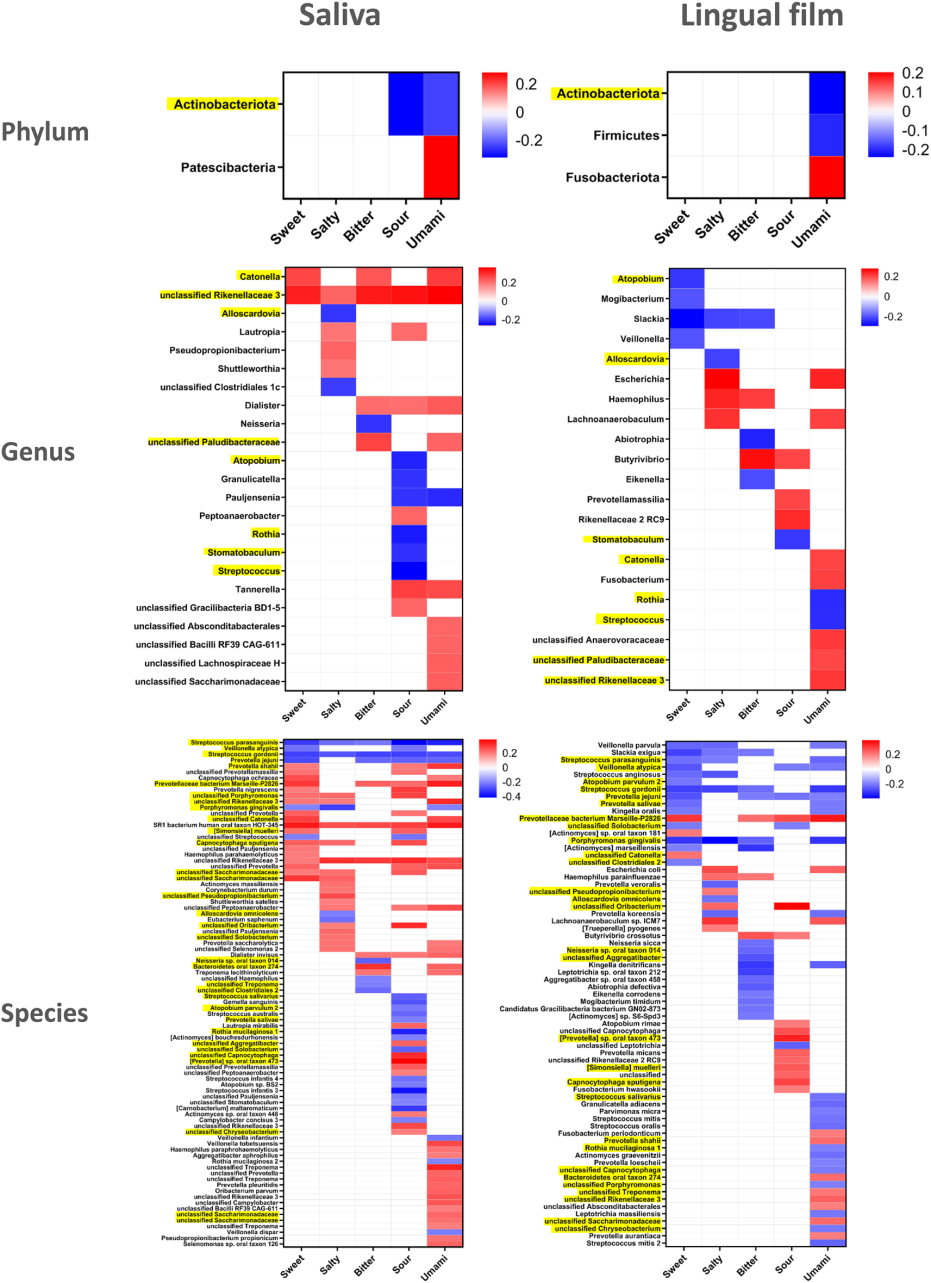
Heatmap showing the Spearman correlation among differential phylum, genus, species expression and taste sensitivity scores in saliva and lingual film. Phyla, genera and species highlighted in yellow are common to the saliva and lingual film. Positive correlations are represented in red, and negative ones in blue. A positive correlation means that a higher abundance of the microbial taxa corresponds to a higher sensitivity to the taste. (*p* value < 0.05).

No significant correlation between the sensitivity to the five tastes and microbial richness (MSP count) was observed for either the saliva or the lingual film (Table 2). Correlations were made between individual taste sensitivity scores and microbes at the phylum, genus and species taxonomic levels, as shown in Fig. 4. Saliva and lingual microbiota are both informative, with more significant correlations in the salivary ecosystem; 86 in saliva species vs. 69 in lingual film, consistent with the higher number of species identified at this site (see above and Fig. 3A). Generally, approximately twice as many positive correlations as negative correlations between species and taste are found in the saliva, whereas the opposite is true in the lingual film. In the saliva, umami and sour tastes display more correlations with microbial taxa than other tastes, i.e., 37 and 44 correlations at the species level, similar to umami in the lingual film (34 correlations). In the saliva, most of the correlations were positive with sweetness (18 positive vs. 6 negative correlations at the species level), saltiness (17 vs. 5) and umami (30 vs. 7), whereas in the lingual film, most of the correlations were positive with sourness (18 vs. 4) and negative with sweetness (3 vs. 14) and bitterness (3 vs. 16). Other combinations are rather evenly distributed. It is worth mentioning that the Spearman coefficient distribution is similar between the different tastes at the genus and species levels but varies at the phylum level, with the highest positive and negative values observed with umami taste in saliva and lingual film data and the highest negative values observed with sour and umami taste in saliva and lingual film, respectively (Supplementary Fig. S1). Note that only the difference between sour and umami is significant (Wilcoxon *p* value = 0.02).

**Table 2:** Spearman correlations and p values associated with correlation tests performed between MSP richness and taste sensitivity.

	Saliva		Lingual film	
	Correlation	*p* value	Correlation	*p* value
Sweet	0.08	0.79	− 0.03	0.79
Salty	0.09	0.46	− 0.07	0.46
Bitter	−0.06	0.33	− 0.10	0.33
Sour	0.09	0.28	0.11	0.28
Umami	0.10	0.63	− 0.05	0.63

**Figure 5:**
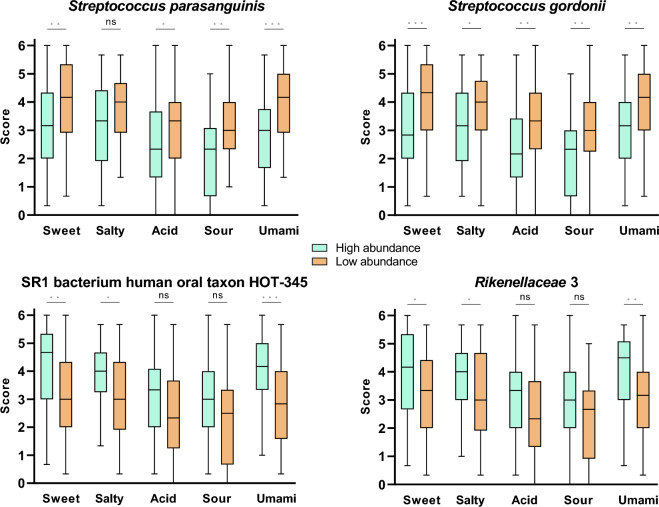
Boxplots showing the distribution of sensitivity scores of the participants according to their high (N = 50) or low (N = 50) abundance in the four species correlated significantly to the sensitivity to the five tastes in saliva. The number of stars (*p < 0.05; **p < 0.01; ***p < 0.001; ns: nonsignificant) indicates the significance level of the Wilcoxon test depicting the difference between the two groups.

Four phyla were significantly associated with umami taste: Fusobacteriota and Patescibacteria were positively correlated, and Actinobacteria and Firmicutes were negatively correlated. Actinobacteria is the only genus appearing in both saliva and film ecosystems and is also negatively correlated with sour taste in the saliva. At the genus and species levels, a large portion of bacterial taxa that correlated with at least one taste sensitivity in the lingual film also correlated with one taste in the saliva (8 genera out of 36 and 46 species out of 109, highlighted in yellow in Fig. 4). Generally, correlations occur in the same direction but not always for the same taste. For instance, *Rothia* and *Streptococcus* genera are negatively correlated with the sour taste in the saliva and with the umami taste in the lingual film. Among the most abundant species in the oral area (> 10^–6^ in saliva or film), *Veillonella atipyca*, *Porphyromonas gingivalis*, *Neisseria* sp. oral taxon 014, *Streptococcus salivariu*s*, Atopobium parvulum* 2, *Prevotella salivae* and *Rothia mucilaginosa* 1 display negative correlations with one or several taste sensitivities in both oral sites, and *Prevotella shahii* and unclassified *Saccharimonadaceae* display positive correlations. While over half of the species correlated with a single taste, some species correlated with four or five tastes, all in the same direction: *Streptococcus parasanguinis, Streptococcus gordonii, P. gingivalis* and *Prevotella jejuni* had negative correlations, and unclassified *Rikenellaceae* 3, SR1 bacterium human oral taxon HOT-345 (SR1 HOT-345), and *Prevotellaceae* bacterium Marseille-P2826 had positive correlations. Hence, *P. gingivalis*, *S. parasanguinis, S. gordonii* and *P. jejuni were* negatively correlated with three or four similar tastes in both saliva and lingual film. In contrast, *Prevotellaceae* bacterium Marseille positively correlated with all tastes except saltiness at both sites. To go beyond correlations, we selected the species that displayed correlations for all tastes in saliva (i.e., *S. parasanguinis, S. gordonii* for negative correlations; unclassified *Rikenellaceae* 3 and SR1 HOT-345 for positive correlations); for each species, the participants were divided into two groups (n = 50) according to the proportion of each species in their saliva microbiota, and the taste scores were compared between the two groups. For the two *Streptococcus* species, taste scores were significantly lower for the group with the highest proportion of the studied species for all the tastes except salty for *S. parasanguinis* (Fig. 5). For SR1 HOT-354 and *Rikenellaceae* 3, sweet, salty, and umami scores were significantly higher for the groups with the highest proportion of the species. Differences between taste scores were also found for the species that correlated with four tastes and the lingual film, but to a lesser extent (Supp. Figure 2). These results validate the tendency highlighted by the correlations.

For most taxa, the genus level recapitulated the direction of the correlation. For example, all selected *Streptococcus* and *Rothia* species showed negative correlations, while all selected *Saccharimonadaceae* species showed positive correlations. Interestingly, all selected *Veillonella* species displayed negative correlations except *Veillonella tobetsuensis*. For *Prevotella*, the direction of correlations is dependent on the species. For example, *Prevotella salivae* is negatively correlated with sweet and sour perceptions, whereas *Prevotella nigrescens* is positively correlated with these tastes.

**Figure 6:**
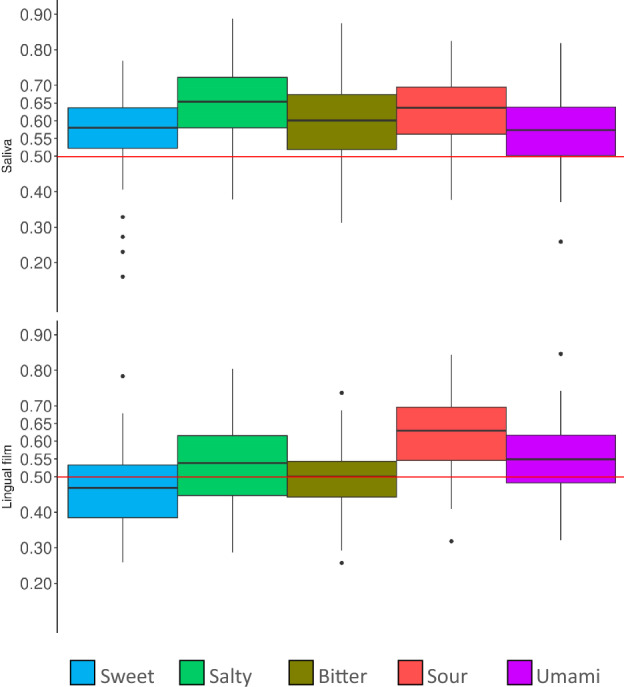
Box plot representation of taste-prediction performances. The upper part of the plot displays the prediction model performances when predicting taste scores with saliva taxa. The lower part displays the prediction model performances when predicting taste scores with lingual taxa.

The impact of a bacterial species does not seem to be linked to its abundance or prevalence since most of the species that correlate with taste sensitivity are not particularly abundant (< 10^–6^) or prevalent (< 70) (Supplementary Table 1). To identify if the microbiota from both oral sites studied can predict taste sensitivity, prediction models performances were determined through AUC computation (see Methods) (Fig. 6). It appears that the saliva presents better taste-predictive capacities than the lingual film. Nevertheless, the predictive scores remain of mediocre quality, with the highest qualities observed when predicting salty or sour sensitivity using saliva microbiome species (mean AUC predictive score = 0.65 and 0.63, respectively).

## Discussion

### Comparison between saliva and lingual film microbiota

The objective of the present work was to elucidate whether oral microbiota composition is related to taste perception. We focused on two ecosystems, the salivary and lingual film microbiota, which is present on the tongue dorsum and cover the taste papillae. The latter is suspected to modulate the concentration of taste compounds in the vicinity of taste receptors^7^, whereas the resting saliva microbiota may reflect the various niches of the oral cavity and can be relevant as a future source of markers. We first identified 666 different species, which is comparable with the current number of 774 oral species reported in the expanded Human Oral Microbiome Database^26^. This represents an important diversity, especially considering that we studied participants from the same living area. The second interesting observation is that most species are significantly more abundant in the saliva than in the lingual film, but the lingual film appears to be characterized by a lower presence of most of the shared species between individuals than that observed in the saliva.

On average, the saliva and lingual film microbiota profiles are characterized by an overrepresentation of the Proteobacteria, Firmicutes and Bacteroidota phyla comparable to the profiles found recently by other authors using metagenomics^27^ and 16S rRNA data from the eHOMD^28–30^. Our study shows that saliva contains more Bacteroidota and less Actinobacteria than lingual film, consistent with our previous results obtained using qPCR on a smaller number of participants^23^. However, the eHOMD data reported this difference in Bacteroidota but not in Proteobacteria, and no differences were observed by Liu et al.^27^. In addition to the different origins of the three panels, US young adults (eHOMD), Chinese^27^ and French (present study) individuals and the different analytic methods, it is worth noting that the sampling protocols differed. For instance, the sampling in the present study consisted of scrapping the tongue dorsum by an experimenter, whereas in the study of Liu et al. the sampling was performed by the participants themselves^31^. Self-sampling by the participants is generally softer than when performed by an experimenter, resulting in sampling of the superficial layer of the lingual microbiota, which may be more heterogeneous. We collected all the microbial material in the tongue dorsum, including microorganisms installed in the deep layers of the lingual film close to the taste receptors.

In addition to the observed specificities in the profiles, saliva and lingual film microbiota vary importantly between individuals, as already reported^18, 27^; however, the causative factors of this variability have not been fully identified. We reported an effect of saliva flow on salivary microbiota. This is not surprising since saliva has antibacterial properties, especially due to its protein composition^26, 27^. This fluid contains various compounds, including glycoproteins and sugars, that can be used as substrates for microorganisms^11^, modulating the microbiota composition differently. Saliva flow also plays a role in cleaning the oral cavity, favoring the unhooking of microorganisms from the various niches of the oral cavity. It is, however, less surprising that the lingual film is not correlated with the saliva flow rate; its microbiota is firmly anchored to the lingual mucosa. Age was also significantly correlated with the lingual film microbiota but not with that of the saliva. This lack of correlation was unexpected since it is known that the microbiota of the different niches of the oral cavity varies with age^32^.

### Oral microbiota and taste

From a global view, microbial richness is not correlated with any specific taste sensitivity in the lingual film or saliva. No correlation with microbial richness was observed with 6-*n*-propylthiouracyl (bitter) perception by Cattaneo et al.^14^ or with linoleic acid (fatty acid) perception by Besnard et al.^13^. Altogether, these observations suggest that the link between taste sensitivity and single taxa must be considered. Moreover, regarding the hypothesis that bacterial metabolites could modulate the concentration of the taste molecules near the taste receptors, we could expect opposite correlations for the same taxon. For example, some saccharolytic and acidogenic bacteria could impact sweet and sour taste perceptions in opposite ways. In the context of this hypothesis, it seemed important to properly test the perception of the 5 tastes simultaneously in all individuals and not a single molecule as a marker of general perception. Surprisingly, no bacterial taxon (regardless of the taxonomic level) correlated positively with one taste and negatively with another. Without rejecting the hypothesis, it could be assumed that active metabolic pathways imply intricate interspecies interactions that cannot be analysed in this study. In contrast, this study highlights some bacterial taxa that correlate in the same direction for several tastes, especially some *Streptococcus* and *Prevotella* species. These observations cannot be made at the genus level, probably because it represents the mean of species with and without correlation with taste perception. This must be why Cattaneo et al.^15^ did not observe correlations using 16S RNA gene sequencing. Our correlations at the genus level do not match exactly with theirs. *Lachnoanaerobaculum*, *Fusobacterium* and *Rothia* are in the same direction but not always for the same taste, and both studies highlight original genera. Comparing with a study from the same group, we only observed one common genus, *Catonella,* correlated to taste^14^. These differences between studies could arise from the various approaches employed, especially the sequencing techniques and the selected populations.

*Streptococcus*, which is the most abundant genus in the saliva and on the tongue, is negatively correlated only with sourness in the saliva and only with umami in the lingual film. This finding was confirmed at a lower taxonomic rank since none of the species of this genus positively correlated with taste. *S. gordonii* and *S. parasanguinis* are remarkable since they are negatively correlated with all taste modalities in the saliva. This example illustrates the importance of considering the species level in such studies, with the genus level being an average of the contribution of species that could have opposite effects. This finding highlights the added value of shotgun metagenomics, which allows quantification at the species level.

Such an implication of *Streptococcus* in taste had already been observed in elderly patients with a link between a global lower taste perception and the abundance of culturable salivary *Streptococci*^8^ and in children with a link between a 6-*n*-propylthiouracil lower taste perception and the abundance of culturable salivary *S. mutans*^33^. *Streptococci* are important primary colonizers of the mouth because of their ability to adhere to tooth surfaces. The nature and number of adhesins vary among *Streptococcus* species, and *S. gordonii* is particularly adept at adhering to various surfaces and coaggregating with various bacterial species due to its numerous adhesins^34, 35^. Wilbert et al.^36^ observed that oral bacterial biofilms on the lingual papillae are organized in patches and that the special organization of the two *Streptococcus* species they studied was different, with *S. salivarius* forming patches and *S. mitis* forming layers at the biofilm surface. We assume that *S. gordonii* and *S. parasanguinis* would impact the structure of the lingual biofilm in a way that increases constraints for the diffusion of the taste molecules to the taste receptors. Moreover, these *Streptococcus* species can compete with other species by producing H_2_O_2_ and bacteriocins, which can also modify the biofilm structure^37^.

The *Prevotella* genus was the second most abundant genus in terms of abundance at both oral sites, consistent with previous studies^38^. The species belonging to this genus are particularly heterogeneous regarding correlations, especially *Prevotellaceae* bacterium Marseille-P2826, for which positive correlations were found for 4 tastes, and *P. jejuni,* which is negatively correlated with 4 tastes. These correlations occur in both oral sites. The physiology and functionality of this genus have been poorly studied to date, mainly because of culturing difficulties. Recent advances in metagenomics have uncovered the large diversity of the *Prevotella* genus, and the oral cavity hosts the largest diversity^38^. The different *Prevotella* species correlating in the same direction are not the closest phylogenetically.

## Conclusion

One of the aims of the present work was to identify key microorganisms from the oral microbiota involved in taste perception by exploring two potentially involved oral ecosystems. We found several correlations between the different taste modalities and various bacterial species, some of which were common to both ecosystems. Our study reveals the importance of 2 main oral bacterial genera for taste perception. The abundance of *Streptococcus* species, especially *S. gordonii* and *S. parasanguinis*, is correlated with a reduced taste sensitivity, whereas the type of correlation is species-dependent for the *Prevotella* genus. When trying to answer the question of which ecosystem is the most linked to taste sensitivity, it appears from our modelling approach that the saliva microbiota is a better taste predictor than that of the lingual film. This result is somewhat surprising since we expected that the lingual microbiota, at the interface between the oral cavity and the taste receptors, would be implied directly in this perception. In this context, saliva appears to be the sum of the microbiota of the various niches of the oral cavity. Therefore, one possible explanation is that the implication of the salivary microbiota is to find at the oral level by studying the global metabolic activity involving species consortia. In the overall context of taste perception, a next step would be to understand the contribution of the oral microbiota alongside other factors involved in taste perception such as the genetics of taste receptors, the number of taste buds or the biochemistry of saliva.

### Supplementary Information

Supplementary Information 1.

Supplementary Information 2.

Supplementary Information 3.

## Data Availability

Raw shotgun sequencing data that support the findings of this study have been deposited in the European Nucleotide Archive with accession codes PRJEB60621 (https://www.ebi.ac.uk/ena/browser/view/PRJEB60621). Correspondence and material request can be addressed to eric.neyraud@inrae.fr.
