# Supplementary material for: The bacterial species profiles of the lingual and salivary microbiota differ with basic tastes sensitivity in human
Source: Sci Rep. 2023 Nov 20;13:20339. doi: 10.1038/s41598-023-47636-1 (PMC10663626; doi:10.1038/s41598-023-47636-1)
Supplement: Supplementary file 1 — Supplementary Information 1. [file 41598_2023_47636_MOESM1_ESM.pptx]

## Slide 1
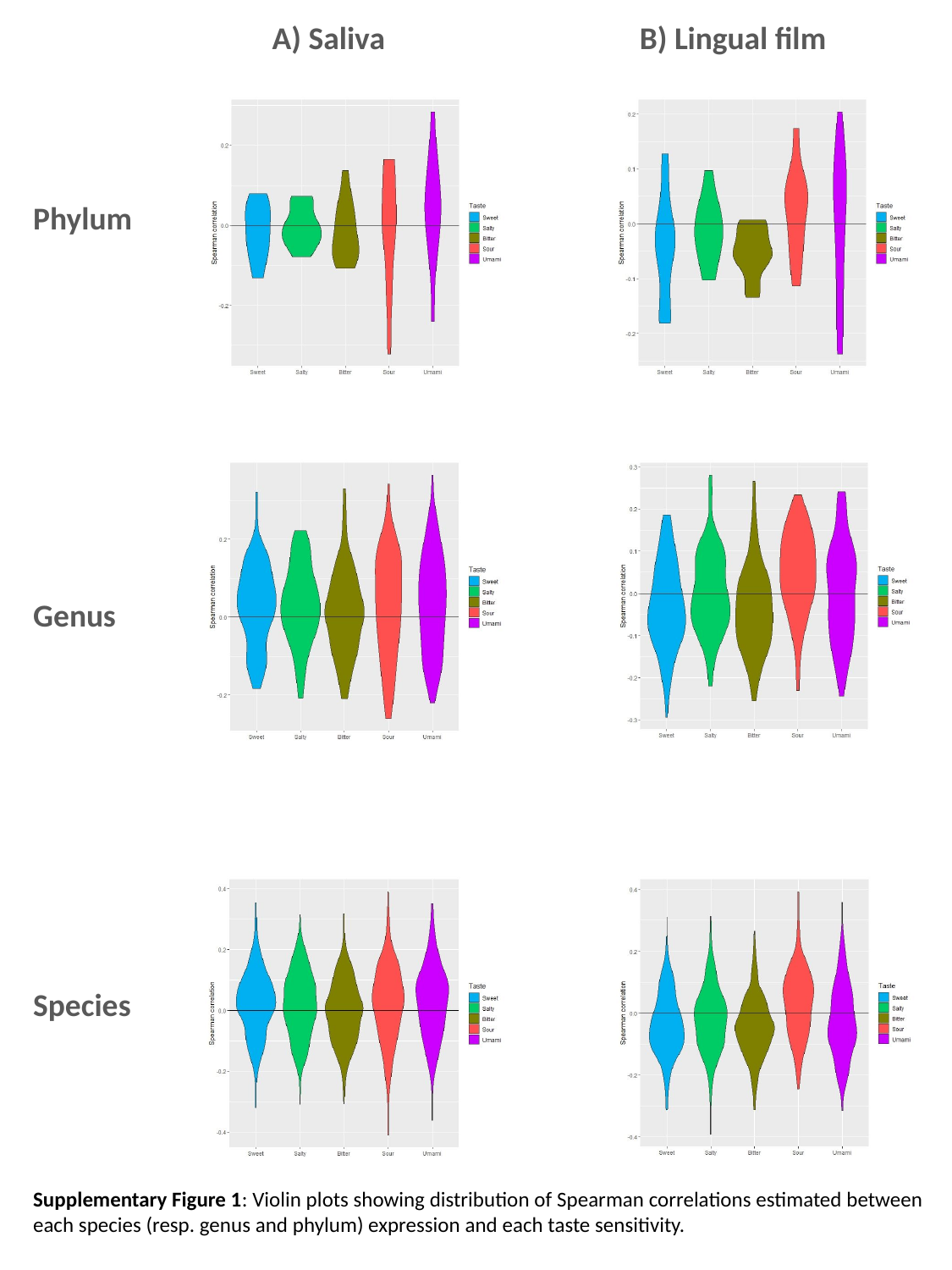

A) Saliva
B) Lingual film
Phylum
Genus
Species
Supplementary Figure 1: Violin plots showing distribution of Spearman correlations estimated between each species (resp. genus and phylum) expression and each taste sensitivity.
