# Supplementary material for: The bacterial species profiles of the lingual and salivary microbiota differ with basic tastes sensitivity in human
Source: Sci Rep. 2023 Nov 20;13:20339. doi: 10.1038/s41598-023-47636-1 (PMC10663626; doi:10.1038/s41598-023-47636-1)
Supplement: Supplementary file 2 — Supplementary Information 2. [file 41598_2023_47636_MOESM2_ESM.pptx]

## Slide 1
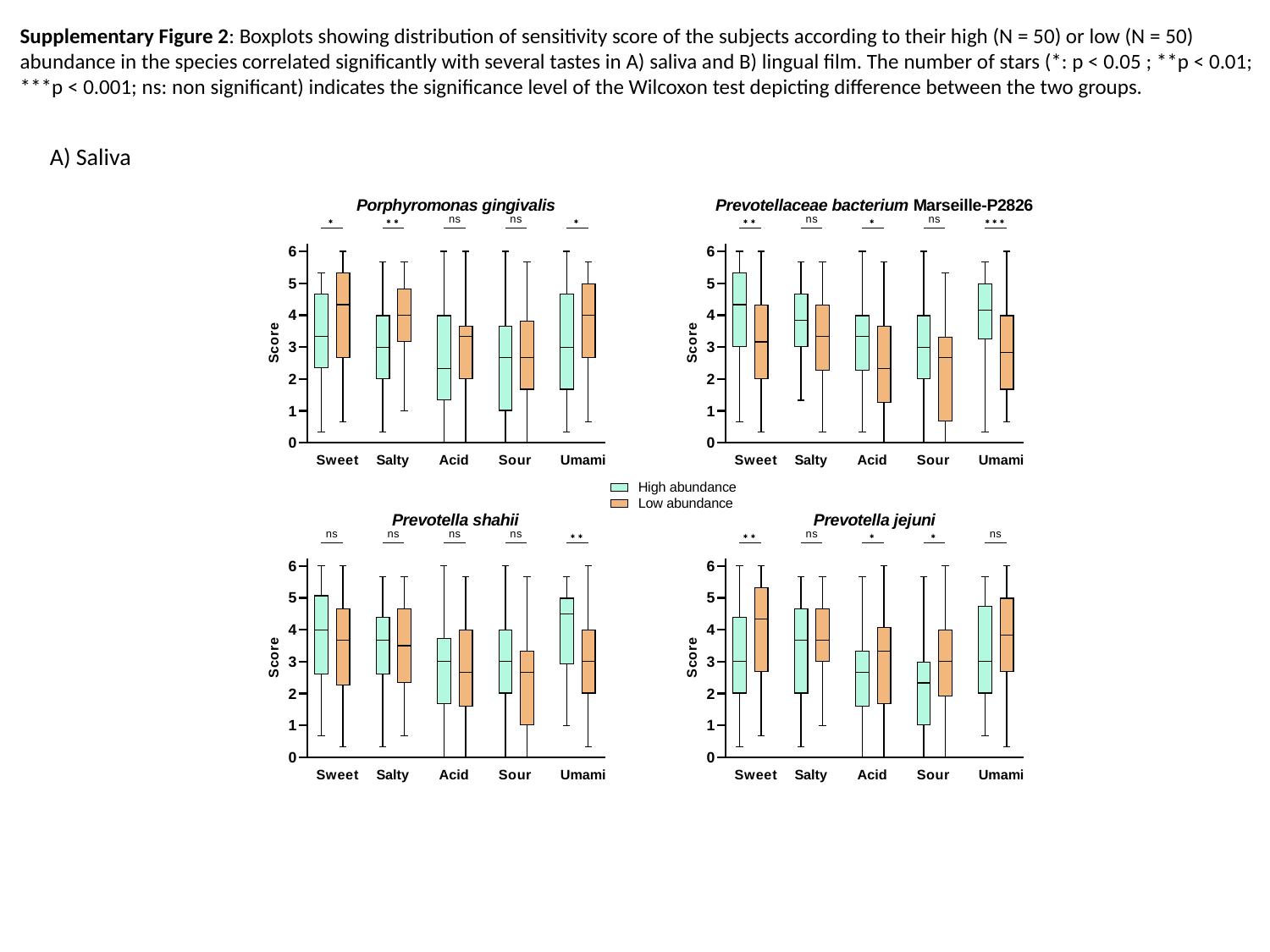

Supplementary Figure 2: Boxplots showing distribution of sensitivity score of the subjects according to their high (N = 50) or low (N = 50) abundance in the species correlated significantly with several tastes in A) saliva and B) lingual film. The number of stars (*: p < 0.05 ; **p < 0.01; ***p < 0.001; ns: non significant) indicates the significance level of the Wilcoxon test depicting difference between the two groups.
A) Saliva

## Slide 2
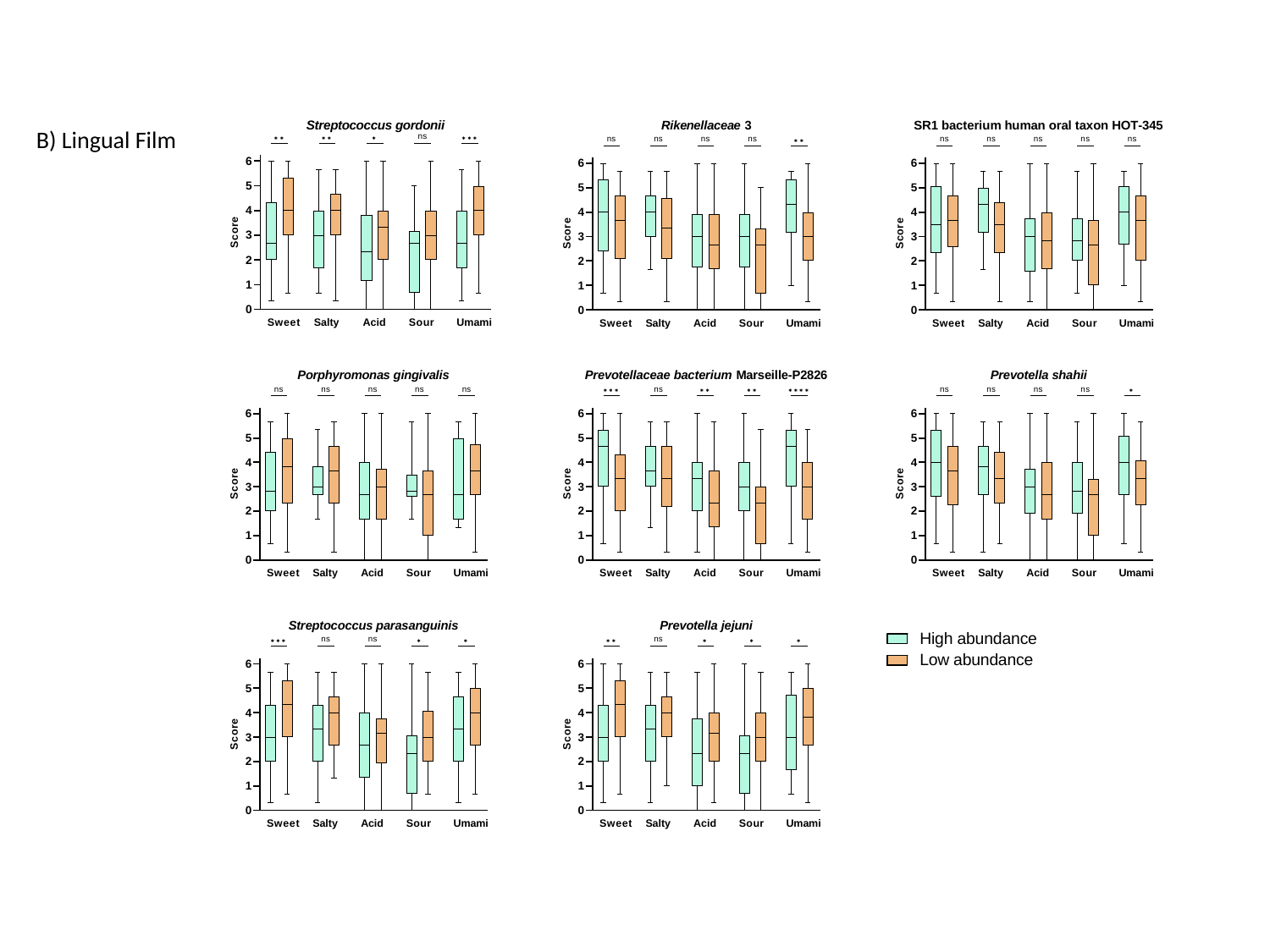

B) Lingual Film
